# Femoral Hernia

**Published:** 1875-07

**Authors:** John McLean

**Affiliations:** DuQuoin, Ill.


Article IV.

### FEMORAL HERNIA.

By JOHN McLEAN, M.D., Du Quoin, Ill.

Mrs. H——, æt. 42, mother of ten children. Attacked Nov. 8, 1874, with cholera morbus. After a few hours the bowels ceased moving. Vomiting continued. On the 9th was seen by a physician, who diagnosed her case one of intussusception, and treated her accordingly, until the 16th. Eight days after the attack, when I was called at his request, I found her with a pulse of 130; respirations, 28; skin cold and clammy, and vomiting stercoraceous matter; abdomen flaccid, and free from pain or soreness upon pressure. But upon an examination of the right femoral region, found a tumor the size of a walnut which I at once diagnosed a femoral hernia, and proceeded to operate as soon as the patient could be brought under the influence of chloroform.

The first thing brought to view was the appendix vermiformis and the adjacent colon and illium, all of which were in a gangrenous condition. Cut away the appendix, and made a longitudinal incision one inch in length in the colon, commencing at the illeo cœcal valve. I then relieved the gut from the constriction, drew the edges of the wound out to the wound in the skin, and attached them with sutures; then passed my finger in each direction of the gut, when fœcal matter came away.

The recovery was rapid, and all the alarming symptoms subsided at once.

The bowels were evacuated through the artificial opening for two weeks, when an evacuation per rectum occurred, followed by others each day. The artificial opening discharged only a watery substance up to about February 1st, 1875, when it ceased entirely to discharge, and is now almost entirely healed, and the woman is in good health.

